# Melatonin improves non-alcoholic fatty liver disease via MAPK-JNK/P38 signaling in high-fat-diet-induced obese mice

**DOI:** 10.1186/s12944-016-0370-9

**Published:** 2016-11-23

**Authors:** Hang Sun, Xingchun Wang, Jiaqi Chen, Kexiu Song, Aaron M. Gusdon, Liang Li, Le Bu, Shen Qu

**Affiliations:** 1 Department of Endocrinology and Metabolism, Shanghai Tenth People’s Hospital, School of Medicine, Tongji University, Shanghai, 200072 China; 2 Nanjing Medical University, Nanjing, Jiangsu 210029 China; 3 Department of Neurology, Weill Cornell Medical College, New York, NY 10065 USA

**Keywords:** Melatonin, NAFLD, Obesity, Inflammation, MAPK

## Abstract

**Background:**

Melatonin can regulate lipid metabolism, increase insulin sensitivity, regulate glucose metabolism and reduce body weight. This study is aimed to determine the effects and mechanism of action of melatonin on non-alcoholic fatty liver disease (NAFLD) in high-fat-diet (HFD) induced obese mice.

**Methods:**

NAFLD was induced by HFD in C57BL/6 mice. A total of 24 mice were randomly assigned to 4 groups. Groups A and B were fed with HFD for 36 weeks while groups C and D were fed with a regular diet (RD). During the last 12 weeks, Groups A and C were treated with 10 mg/kg melatonin while Groups B and D were treated with water in the same volume by intragastric administration. Body and liver weight, blood glucose, serum transaminases and lipid levels, and markers of hepatic inflammation were measured. Histological analyses were also performed on liver tissue.

**Results:**

After 12 weeks of treatment with melatonin, body weights (Group A: before 53.11 ± 0.72 *vs after 12w treatment* 39.48 ± 0.74) and liver weights (A 1.93 ± 0.09 g vs B 2.92 ± 0.19 g vs C 1.48 ± 0.09 g vs D 1.49 ± 0.10 g), fasting plasma glucose, alanine transaminase (A 24.33 ± 11.90 IU/L vs B 60.80 ± 10.18 IU/L vs C 13.01 ± 3.49 IU/L vs D 16.62 ± 2.00 IU/L), and low-density cholesterol (A 0.24 ± 0.06 mmol/L vs B 1.57 ± 0.10 mmol/L vs C 0.28 ± 0.06 mmol/L vs D 0.29 ± 0.03 mmol/L) were significantly decreased in HFD mice. HFD fed mice treated with melatonin showed significantly less liver steatosis. Treatment of HFD fed mice with melatonin led to a significant decrease in the expression of TNF-α, IL-1β, and IL-6 measured using quantitative real-time polymerase chain reaction (qRT-PCR). HFD fed mice demonstrated increased phosphorylation of P38 and JNK1/2, which was reduced by melatonin treatment.

**Conclusions:**

The study concluded that melatonin could improve NAFLD by decreasing body weight and reduce inflammation in HFD induced obese mice by modulating the MAPK-JNK/P38 signaling pathway.

## Background

Non-alcoholic fatty liver disease (NAFLD) is characterized by fat accumulation in hepatocytes and precedes hepatic steatosis and occurs in the absence of alcohol consumption [1]. NAFLD is a growing public health concern worldwide because of its high morbidity and its association with hyperlipidemia, diabetes, and obesity [1, 2]. Simple steatosis occurs early in the course of NAFLD and progresses to nonalcoholic steatohepatitis (NASH) and finally to liver cirrhosis [3]. Obesity is an important risk factor driving the development of NAFLD [4]. A high-fat-diet (HFD) may act as the ‘first hit’ leading to NAFLD while inflammation likely serves as a ‘second hit’ driving progression from NAFLD to NASH [5]. Currently, treatment for NAFLD is mainly aimed at modifying risk factors, but there are no therapies directly targeting underlying liver pathology [6].

Melatonin is a hormone secreted by the pineal gland. The synthesis and secretion of melatonin are regulated by light intensity [7]. It has been demonstrated that melatonin has multiple biological effects and acts as an anti-oxidant [8, 9]. It has anti-inflammatory properties [10, 11], regulates circadian rhythms [12] and immunity [13]. It also has anti-neoplastic effects [14]. Recent research has also found that melatonin can regulate lipid metabolism [15], increase insulin sensitivity [16], regulate glucose metabolism [17], and reduce body weight [18]. It was found that melatonin could improve HFD-induced NAFLD and attenuate oxidative stress [19]. Stacchiotti *et al.* also found that melatonin treatment could improve hepatic macrosteatosis in NAFLD in *ob/ob* mice [20]. However, the underlying mechanisms remain unclear. This study was designed to investigate the effect of melatonin on HFD induced NAFLD and explore the underlying mechanism.

## Methods

### Experimental design

24 male C57BL/6 mice (6 weeks old, 21.32 ± 0.85 g) were purchased from Shanghai SLAC Laboratory Animal Co. Ltd (Shanghai, China). The mice were housed in a clean room maintained at 24 ± 2 °C under a 12 h:12 h light:dark cycle, with free access to food and water. All animal experiments were approved by the Animal Care and Use Committee of Tongji University with the Ethics Protocol number QS20140305. Mice were randomly assigned to 4 groups. Group A (*n* = 6) and Group B (*n* = 6) were fed with HFD for 36 weeks, while Group C and Group D were fed with regular diet (RD). After 24 weeks, Group A and Group C received 10 mg/kg melatonin by intragastric administration while Group B and Droup D received double-distilled water in same volume by intragastric administration consistently between 7 pm and 8 pm for 12 weeks, according to previous studies [19, 21–23]. HFD and RD were purchased from Meidisen Biomedical Company (Jiangsu, China). The diets follow the AIN93 recommendations. The high fat diet (research diet D12492) contains 60% calories from fat, 20% from carbohydrates and 20% from proteins. The regular diet (research diet D12450B) contains 10% calories from fat, 70% from carbohydrates and 20% from proteins. Melatonin was purchased from Sigma Aldrich Chemical Co. (St. Louis, MO, USA), dissolved in water. The ethanol concentration in the final solution was 1%. At the end of the experimental time period, mice were anaesthetized using pentobarbital sodium, blood was collected via cardiac puncture, and livers were removed upon sacrifice.

### Body weight and liver weight evaluation

Body weight was measured every 4 weeks consistently between 7 pm and 8 pm. Liver weight was measured after sacrifice.

### Blood glucose, transaminase and lipid levels

Blood glucose was determined after tail-snip and measured using a glucose test strip reader. Serum levels of alanine aminotransferase (ALT) and aspartate aminotransferase (AST), triglycerides (TG), low-density cholesterol (LDL-C) and high-density cholesterol (HDL-C) were measured spectrophotometrically according to the instructions (NJJCBIO, Jiangsu, China).

### Histological analyses

Paraffin sections of liver, 4 μm thick, were processed for hematoxylin-eosin (HE) staining. Frozen sections of liver, 8 μm thick, were processed routinely for Oil Red O staining.

Volume density (Vv) of hepatic steatosis was analyzed using the software Image Pro Plus, version 7.01 for Windows (Media Cybernetics, Silver Springs, MD, USA) as described [24].

### Real-time reverse-transcriptase polymerase chain reaction (qRT–PCR)

Total mRNA from the mouse liver was isolated using the Trizol reagent from Invitrogen (Carlsbad, CA). Two micrograms of total RNA were used for cDNA synthesis using an RNeasy kit (QIAGEN) according to the manufacturer's instructions. Quantitative real-time SYBR Green quantitative RT-PCR was performed using a 7900HT real-time PCR system (ABI, CA, USA) to determine the expression of target genes according to the instructions of the SYBR Premix EX Taq (TaKaRa Biotechnology, China). Data were normalized to glyceraldehyde 3-phosphate dehydrogenase (GAPDH) mRNA as an endogenous control and analyzed using the ΔΔCt method. Primer sequences are provided in Table 1.

**Table 1:** Primer sequence of Real-time PCR

Primer Name	Sequence (5′-3′)	Length
TNF-a	CCCTCACACTCAGATCATCTTCT	23
	GCTACGACGTGGGCTACAG	19
IL-1b	GCAACTGTTCCTGAACTCAACT	22
	ATCTTTTGGGGTCCGTCAACT	21
IL-6	TAGTCCTTCCTACCCCAATTTCC	23
	TTGGTCCTTAGCCACTCCTTC	21
IL-10	GCTCTTACTGACTGGCATGAG	21
	CGCAGCTCTAGGAGCATGTG	20
JNK	AGCAGAAGCAAACGTGACAAC	21
	GCTGCACACACTATTCCTTGAG	22
IKKβ	ACAGCCAGGAGATGGTACG	19
	CAGGGTGACTGAGTCGAGAC	20
GAPDH	AGGTCGGTGTGAACGGATTTG	21
	TGTAGACCATGTAGTTGAGGTCA	23

### Western blotting

Expression of total c-Jun N-terminal kinases (JNK), phospho-JNK (p-JNK), total P38 and phospho-P38 (p-P38) in liver were measured by Western blotting as described [25]. Proteins were separated on 12.5% SDS-PAGE gels and transferred onto nitrocellulose membranes. Antibodies were purchased from Cell Signaling Technology (USA). P38, p-P38, JNK, p-JNK expression was analyzed using a rabbit monoclonal antibody (1: 1000) primary antibody, and a rabbit-anti-mouse IgG-HRP (1: 1000) secondary antibody. GAPDH (1: 1000 dilution for the primary antibody and 1: 1000 dilution for the secondary antibody was used as an endogenous control. Western blot bands were scanned and quantified by densitometry analysis using an image analyzer Quantity One System (Odyssey, USA).

### Statistical evaluation

Statistical analyses were performed using SPSS 21.0 software (Chicago, IL) and GraphPad Prism software (GraphPad Software Inc). Data were presented as mean values ± standard errors. The ANOVA test was used to determine differences between groups comparing the internal variability of group with the variability among all experimental groups. Multiple comparisons between the groups were performed using S-N-K method as a post-hoc test. *P* values <0.05 were considered statistically significant. All assays were performed in triplicate.

## Results

In this study, HFD mice treated with melatonin were compared with HFD mice treated with water, and with lean mice being either treated or not treated with melatonin. Lean mice that were treated or not treated with melatonin had similar weights, glucose metabolism, lipid metabolism, and levels of inflammatory markers, so both groups are referred to as controls.

### Body weight and liver mass

**Fig. 1:**
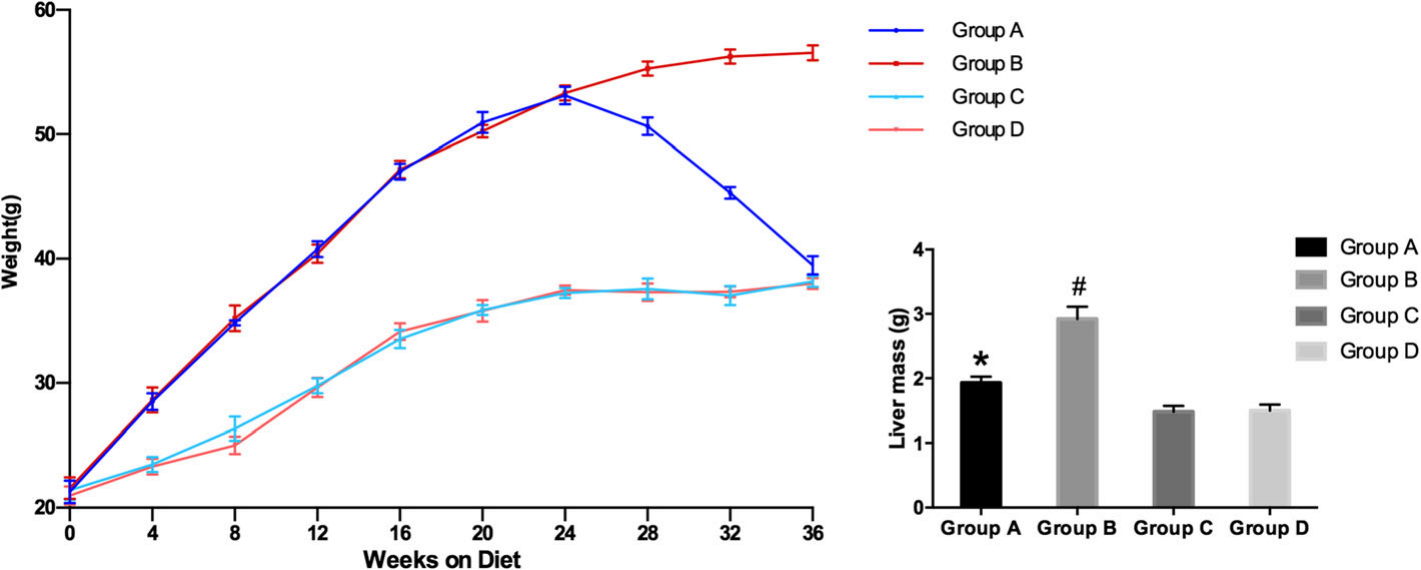
Effect of melatonin on body weight and liver mass. (A) Body weight (g) of HFD with melatonin, HFD, RD with melatonin and RD during 36 weeks, *n* = 6 mice/group. (B) Liver mass of HFD with melatonin, HFD, RD with melatonin and RD at the end of treatment, *n* = 6 mice/group. *P < 0.05 for group A *vs* group B; #*P* < 0.05 for group B *vs* group D

As shown in Fig. 1A, HFD increased body weight and liver mass compared with RD. [at 24w: (A) 53.11 ± 0.72 *vs* (B) 53.30 ± 0.60 *vs* (C) 37.25 ± 0.40 *vs* (D) 37.50 ± 0.39 g]. The average body weight of high fat diet fed mice is ~42.4% more than the regular diet fed mice. Administration of melatonin significantly reduced both body weight by ~30.2% less than the HFD mice [at36w: (A) 39.48 ± 0.74 *vs* (B) 56.56 ± 0.62 *vs* (C) 38.18 ± 0.45 *vs* (D) 38.01 ± 0.44 g]. Livers were removed upon sacrifice and liver mass was measured. As shown in Fig. 1B, the liver mass of melatonin treated mice is ~ 33.9% less than the HFD mice [(A) 1.93 ± 0.09 *vs* (B) 2.92 ± 0.19 *vs* (C) 1.48 ± 0.09 *vs* (D) 1.49 ± 0.10 g] (p < 0.01 between A and B).

### Glucose and lipid metabolism

**Fig. 2:**
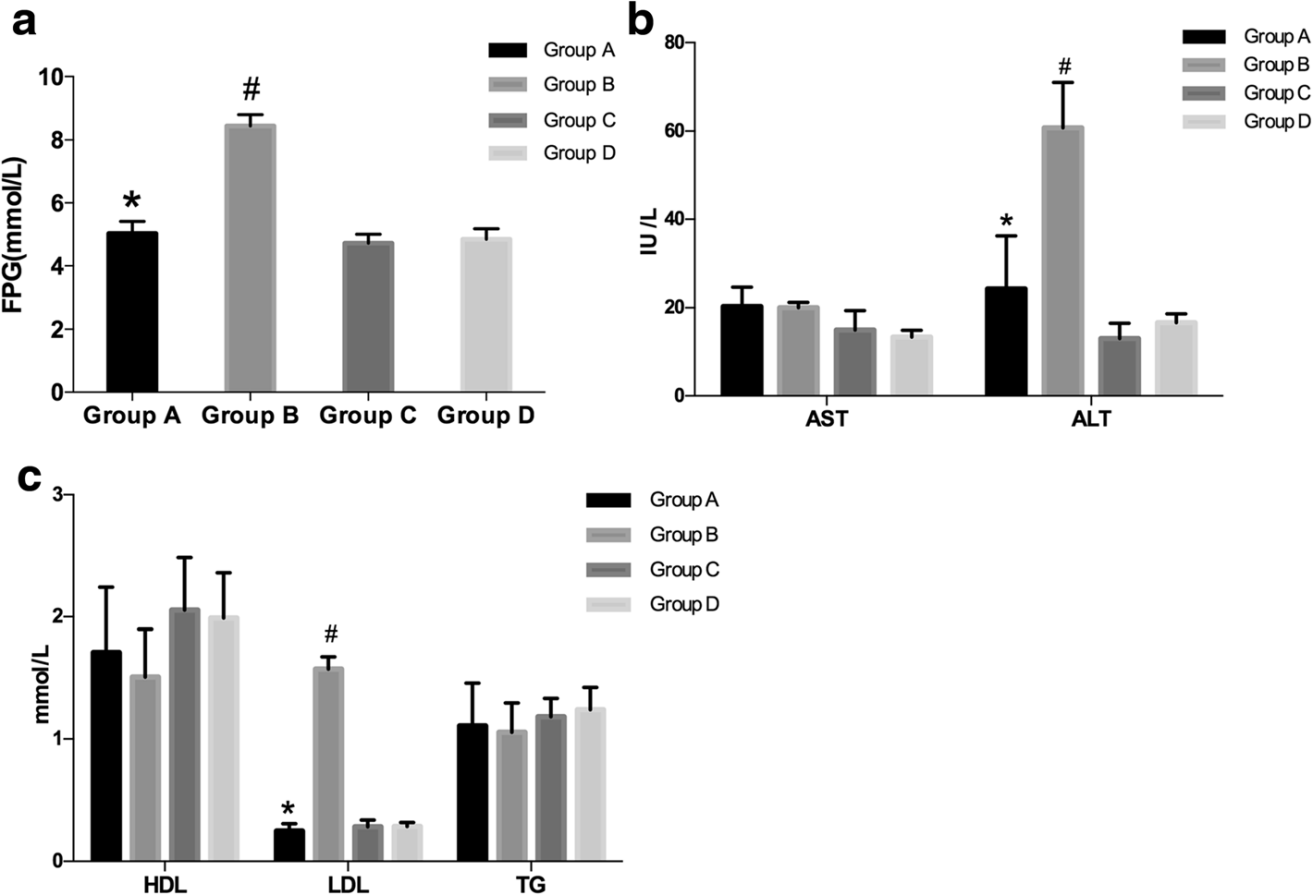
Effect of melatonin on fasting plasma glucose, serum transaminase, and lipid levels. (**a**) Fasting plasma glucose (FPG, mmol/L), *P < 0.05 *vs* group A. (**b**) Alanine aminotransferase (ALT, IU/L) and aspartate aminotransferase (AST, IU/L). *P < 0.05 *vs* other 3 groups. (**c**) Triglycerides (TG, mmol/L), low-density cholesterol (LDL-C, mmol/L) and high-density cholesterol (HDL-C, mmol/L). *P < 0.05 for group A *vs* group B; #*P* < 0.05 for group B *vs* group D

As shown in Table 2 and Fig. 2, after ANOVA test performed among four groups and independent sample T-test used between two groups as a post-hoc test with ANOVA test. It was foundthat melatonin treatment significantly reduced fasting plasma glucose (FPG) (Fig. 2a), ALT (Fig. 2b), and LDL (Fig. 2c) in Group A compared with Group B fed with HFD only, p < 0.01 between A and B.

**Table 2:** Effects of melatonin on fasting plasma glucose, serum transaminase, and lipid levels

	Group A	Group B	Group C	Group D
FPG, mmol/L	5.03 ± 0.38*	8.45 ± 0.35#	4.71 ± 0.29	4.85 ± 0.33
HDL, mmol/L	1.71 ± 0.53	1.51 ± 0.39	2.05 ± 0.43	1.99 ± 0.37
LDL, mmol/L	0.24 ± 0.06*	1.57 ± 0.10#	0.28 ± 0.06	0.29 ± 0.03
TG, mmol/L	1.11 ± 0.35	1.06 ± 0.24	1.18 ± 0.15	1.24 ± 0.18
AST, IU/L	20.31 ± 4.41	20.01 ± 1.20	14.98 ± 4.39	13.35 ± 1.57
ALT, IU/L	24.33 ± 11.90*	60.80 ± 10.18#	13.01 ± 3.49	16.62 ± 2.00

Group A: HFD + MLT, Group B: HFD, Group C: RD + MLT, Group D: RD. *FPG* fasting plasma glucose*, HDL* high-density cholesterol*, LDL* low-density cholesterol*, TG* Triglycerides*, AST* aspartate aminotransferase *and ALT* Alanine aminotransferase. *P < 0.05 for group A vs group B; #P < 0.05 for group B vs group D

### Histological analyses of liver

**Fig. 3:**
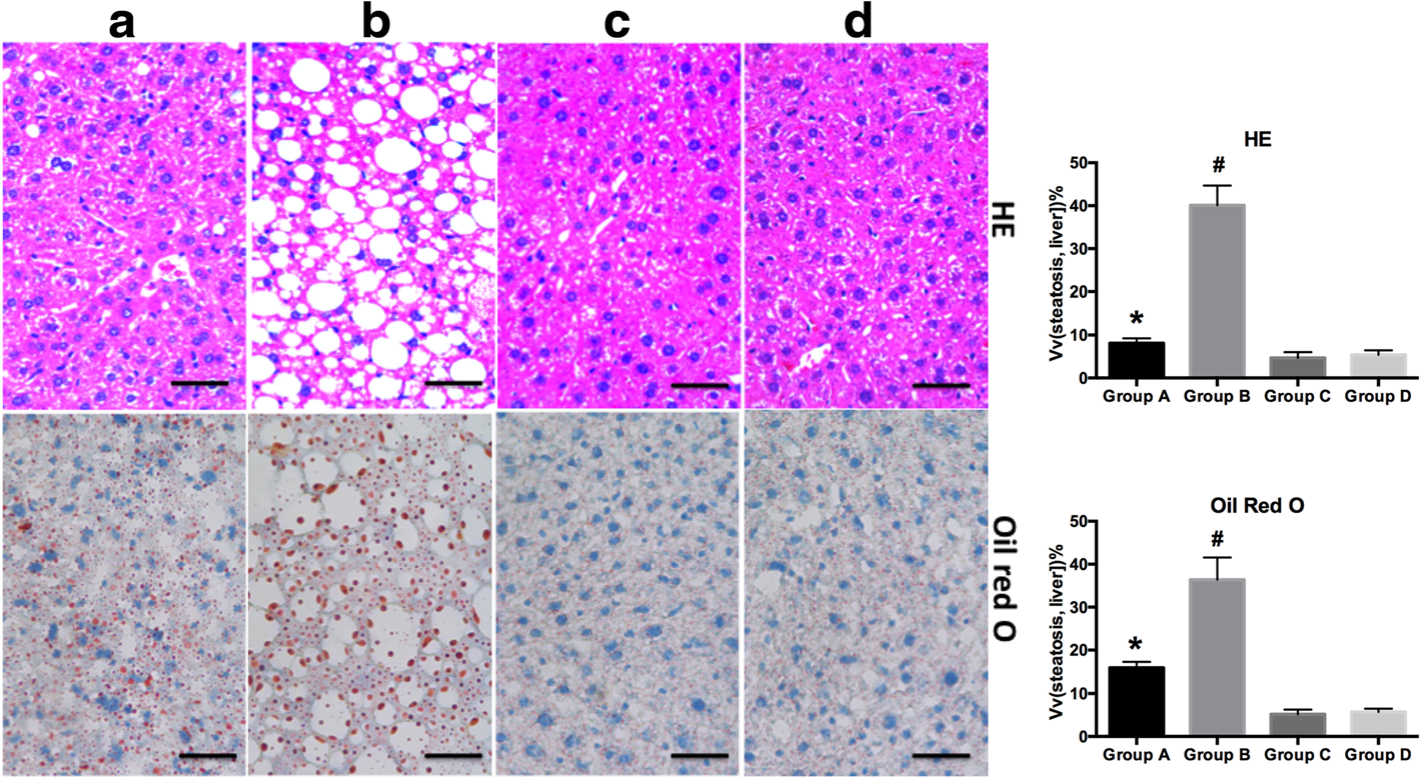
Histological analyses of liver steatosis. Representative images (400×) of H&E-stained (Upper) and Oil Red O-stained (Lower) liver sections from (**a**) HFD with melatonin, (**b**) HFD, (**c**) RD with melatonin, and (**d**) RD

HFD fed mice developed liver steatosis. By contrast, HFD mice treated with melatonin showed a significantly less liver steatosis (Fig. 3).

### Inflammatory

**Fig. 4:**
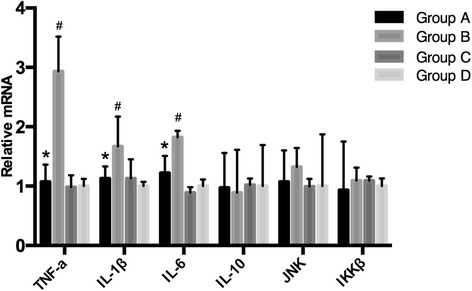
Quantification of pro-inflammatory cytokines. qRT-PCR was conducted in order to analyze the expression of tumor necrosis factor-α (TNF-α), interleukin-1β (IL-1β), interleukin-6 (IL-6), interleukin-10 (IL-10), c-Jun N-terminal kinases (JNK), and inhibitor of nuclear factor kappa-B kinase subunit beta (IKK-β). *P < 0.05 for group A *vs* group B; #*P* < 0.05 for group B *vs* group D

The mRNA levels of pro-inflammatory cytokines including tumor necrosis factor-α (TNF-α), IL-1β, and IL-6 significantly increased in the HFD group compared with RD fed mice, and HFD mice treated with melatonin demonstrated significantly lower expression of TNF-α, IL-1β, and IL-6 (Fig. 4). IL-10 and IKK-β were not significantly different.

**Fig. 5:**
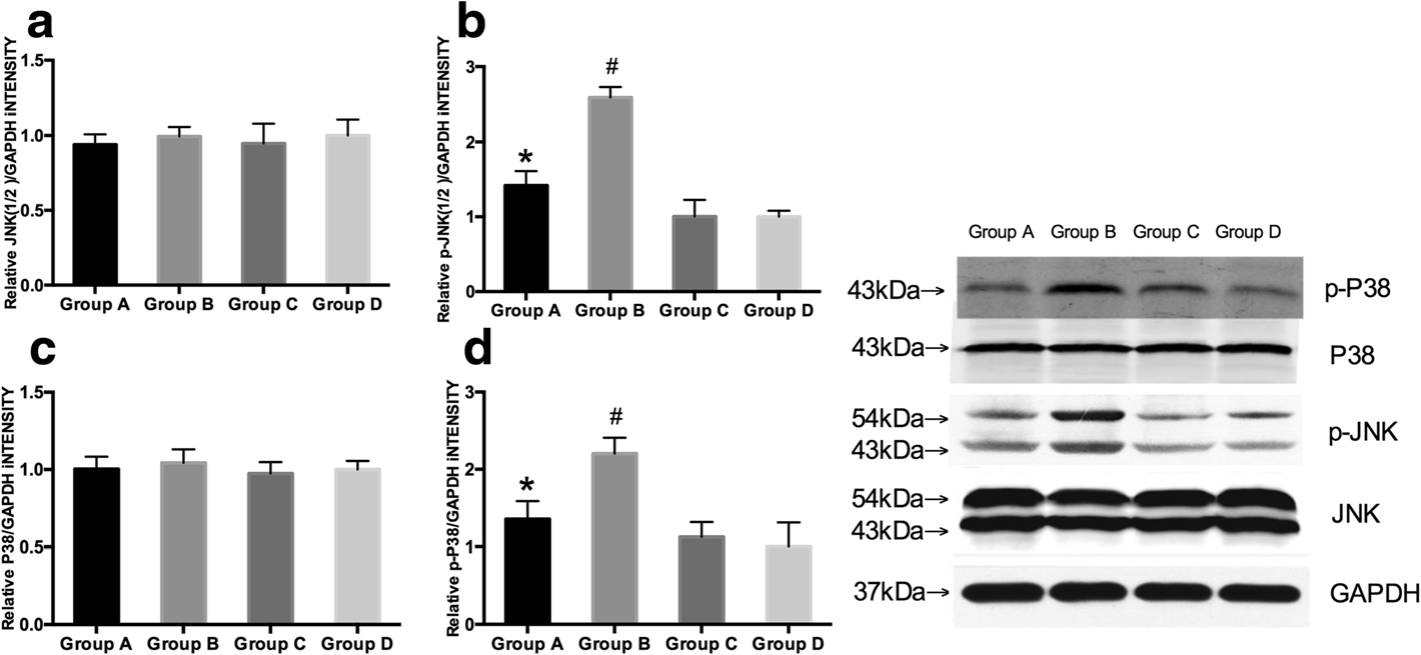
Effect of melatonin on the MAPK-JNK/P38 signaling pathway. Western blot analysis for total and phosphorylated c-Jun N-terminal kinases (JNK) 1/2 and P38, *n* = 6/group. **a** relative JNK(1/2 )/GAPDH intensity of  four groups; **b** relative p-JNK(1/2 )/GAPDH intensity of  four groups; **c** relative P38/GAPDH intensity of  four groups; **d** relative p-P38/GAPDH intensity of four groups; GAPDH: glyceraldehyde 3-phosphate dehydrogenase. *P < 0.05 for group A *vs* group B; #*P* < 0.05 for group B *vs* group D

Furthermore, we evaluated the expression of P38 and c-Jun N-terminal kinases (JNK) 1/2 in liver by Western blotting (Fig. 5). HFD increased phosphorylated levels of P38 and JNK1/2, which was reversed by treatment with melatonin.

## Discussion

Numerous studies have demonstrated that melatonin treatment reduces body weight in both animal models and human [26–29]. A recent study demonstrated that melatonin has effects on inflammation, fat deposition, and energy metabolism in ob/ob mice [30]. In this study, we found that melatonin treatment for 12 weeks significantly decreased body weight and liver weight in HFD mice. Melatonin was also found to regulate glucose metabolism. There are two membrane receptors of melatonin called MT1 and MT2, which are G-protein-coupled receptors, in the periphery [31]. Both receptors are expressed in the islet of Langerhans and are involved in the regulation of glucagon secretion from α-cells and insulin secretion from β-cells [16, 32]. Several studies indicate that activation of MT1 or MT2 leads to a reduction in second messenger 3’,5’-cyclic adenosine monophosphate (cAMP) or 3’,5’-cyclic guano-sine monophosphate (cGMP) accompanied by reduced insulin secretion [33, 34]. Consistent with this mechanism, we found that melatonin treatment significantly reduced HFD induced elevations in blood glucose.

Early experiments showed that treatment with melatonin can improve dyslipidemia [15]. In patients with NAFLD, treatment with melatonin (2-5 mg/day) for 14 months significantly reduced levels of triglycerides (TG) and low density lipoprotein cholesterol (LDL-C) compared with controls treated with Essentiale [35]. Treatment with melatonin for two weeks significantly reduced free fatty acids (FFA) compared with placebo in cigarette smokers [36]. A study assessing aluminium-induced toxicity in a rat model found that melatonin protected against toxic dyslipidemia by alleviating the aluminum induced increases in total cholesterol (TC), LDL-C, TG, oxidized LDL, and apolipoprotein B100 (apoB100) [37]. Pan et al [38] found that melatonin ameliorates NAFLD by lowering serum AST, ALT, liver total cholesterol, and triglycerides in high-fat diet fed rats. Consistently, in this study, we have demonstrated that melatonin administration decreases serum LDL and ALT and reduces the degree of hepatocyte steatosis.

To explore the possible mechanism of melatonin’s beneficial effects on NAFLD, we measured mRNA levels of pro-inflammatory makers and the active levels of mitogen-activated protein kinase (MAPK) signaling. Inflammation contributes to the progression of NAFLD to NASH [5]. We found that melatonin treatment significantly reduced the mRNA level of pro-inflammatory cytokines including TNF-α, IL-1β, and IL-6, which were increased in HFD fed mice. The expression of total JNK1/2 and P38 were similar in four groups, but the phosphorylated levels of P38 and JNK1/2 were increased in HFD mice compared with RD fed mice. The activation of the JNK1/2 and P38 signaling molecules by HFD was reduced by melatonin administration. There are many signaling pathways involved in the progression of hepatic steatosis with numerous interactions among the different pathways. The MAPK signaling pathway is involved in the regulation of inflammation and fatty acid metabolism [39, 40]. HFD leads to inflammation and weight gain, and melatonin treatment could alleviate the inflammation as well as obesity caused by HFD. RD did not lead to any disorders, so melatonin supplemental in RD fed mice would have limited effects on changes in weight, glucose metabolism, lipid metabolism, and inflammation status. Taken together, our data indicate that melatonin affects the signaling pathways leading to elevated inflammatory responses induced by HFD. We hypothesize that the decreased levels of inflammation after melatonin treatment could improve liver pathology in NAFLD and inhibits progression to NASH.

This study had several limitations. Firstly, we did not record the food intake of experimental animals, which was one of the limitations of the study. However, a previous study showed that melatonin treatment could reduce body weight without affecting food intake [41]. And another study demonstrated that ob/ob mice with melatonin administration consumed more food than their genotype counterparts without gaining weight [26]. The effect of melatonin on food intake does need to be further explored.

Reduced body mass could lead to a reduction of adiposity and ectopic accumulation of fat, thus reducing hepatic steatosis. A reduction of steatosis, in turn, down-regulates inflammatory markers. In our study, body mass decreased with melatonin treatment, which could be explained possibly by the melatonin effects on body mass, or other beneficial effects. However, we would also consider the therapeutic effect of melatonin on NAFLD as a result of both anti-inflammation and anti-obesity functions. Previous studies have shown the anti-inflammatory properties of melatonin [10, 11]. In our study, we also found the anti-inflammatory effects on mice fed with HFD, but not on mice fed with regular diet. During the initiation and progression of hepatic steatosis. The dysregulation of lipid metabolism is frequently associated with an inflammatory condition [42], and the elevated inflammatory status plays an important role in the development of insulin resistance, which in turn further promotes ectopic fat accumulation in the liver, forming a vicious cycle [43].

## Conclusion

This study concluded that the treatment with melatonin could decrease body weight and ameliorate liver pathology due to NAFLD in HFD induced obese mice. Melatonin likely decrease inflammation via the MAPK-JNK/P38 signaling pathway.

## Abbreviations

NAFLD: Non-alcoholic fatty liver disease
HFD: high-fat-diet
RD: regular diet
qRT-PCR: quantitative real-time polymerase chain reaction
NASH: nonalcoholic steatohepatitis
ALT: alanine aminotransferase
AST: aspartate aminotransferase
TG: triglycerides
LDL-C: low-density cholesterol
HDL-C: high-density cholesterol
HE: hematoxylin-eosin
JNK: c-Jun N-terminal kinases
p-JNK: phospho-JNK
p-P38: total P38 and phospho-P38
cAMP: 3′,5′-cyclic adenosine monophosphate
cGMP: 3′,5′-cyclic guano-sine monophosphate
FFA: free fatty acids
apoB100: apolipoprotein B100
MAPK: mitogen-activated protein kinase

## References

[1] Rinella ME (2015). Nonalcoholic fatty liver disease: a systematic review. JAMA.

[2] Williams CD, Stengel J, Asike MI, Torres DM, Shaw J, Contreras M, Landt CL, Harrison SA (2011). Prevalence of nonalcoholic fatty liver disease and nonalcoholic steatohepatitis among a largely middle-aged population utilizing ultrasound and liver biopsy: a prospective study. Gastroenterology.

[3] Yeh MM, Brunt EM (2007). Pathology of nonalcoholic fatty liver disease. Am J Clin Pathol.

[4] Cohen JC, Horton JD, Hobbs HH (2011). Human fatty liver disease: old questions and new insights. Science.

[5] Day CP, James OF (1998). Steatohepatitis: a tale of two “hits”?. Gastroenterology.

[6] Marchesini G, Petta S, Dalle Grave R (2015). Diet, weight loss, and liver health in nonalcoholic fatty liver disease: Pathophysiology, evidence, and practice.

[7] Skene DJ, Arendt J (2006). Human circadian rhythms: physiological and therapeutic relevance of light and melatonin. Ann Clin Biochem.

[8] Zhang HM, Zhang Y (2014). Melatonin: a well-documented antioxidant with conditional pro-oxidant actions. J Pineal Res.

[9] Manchester LC, Coto-Montes A, Boga JA, Andersen LP, Zhou Z, Galano A, Vriend J, Tan DX, Reiter RJ (2015). Melatonin: an ancient molecule that makes oxygen metabolically tolerable. J Pineal Res.

[10] Mauriz JL, Collado PS, Veneroso C, Reiter RJ, Gonzalez-Gallego J (2013). A review of the molecular aspects of melatonin's anti-inflammatory actions: recent insights and new perspectives. J Pineal Res.

[11] Borges Lda S, Dermargos A, da Silva Junior EP, Weimann E, Lambertucci RH, Hatanaka E (2015). Melatonin decreases muscular oxidative stress and inflammation induced by strenuous exercise and stimulates growth factor synthesis. J Pineal Res.

[12] Vriend J, Reiter RJ (2015). Melatonin feedback on clock genes: a theory involving the proteasome. J Pineal Res.

[13] Lee JS, Cua DJ (2015). Melatonin Lulling Th17 cells to sleep. Cell.

[14] Di Bella G, Mascia F, Gualano L, Di Bella L (2013). Melatonin anticancer effects: review. Int J Mol Sci.

[15] Sun H, Huang FF, Qu S (2015). Melatonin: a potential intervention for hepatic steatosis. Lipids Health Dis.

[16] Peschke E, Bahr I, Muhlbauer E (2013). Melatonin and pancreatic islets: interrelationships between melatonin, insulin and glucagon. Int J Mol Sci.

[17] Espino J, Pariente JA, Rodriguez AB (2011). Role of melatonin on diabetes-related metabolic disorders. World J Diabetes.

[18] Srinivasan V, Ohta Y, Espino J, Pariente JA, Rodriguez AB, Mohamed M, Zakaria R (2013). Metabolic syndrome, its pathophysiology and the role of melatonin. Recent Pat Endocr Metab Immune Drug Discov.

[19] Hatzis G, Ziakas P, Kavantzas N, Triantafyllou A, Sigalas P, Andreadou I, Ioannidis K, Chatzis S, Filis K, Papalampros A, Sigala F (2013). Melatonin attenuates high fat diet-induced fatty liver disease in rats. World J Hepatol.

[20] Stacchiotti A, Favero G, Lavazza A, Golic I, Aleksic M, Korac A, Rodella LF, Rezzani R (2016). Hepatic Macrosteatosis is partially converted to microsteatosis by melatonin supplementation in ob/ob mice non-alcoholic fatty liver disease. PLoS One.

[21] Jimenez-Aranda A, Fernandez-Vazquez G, Campos D, Tassi M, Velasco-Perez L, Tan DX, Reiter RJ, Agil A (2013). Melatonin induces browning of inguinal white adipose tissue in Zucker diabetic fatty rats. J Pineal Res.

[22] Navarro-Alarcon M, Ruiz-Ojeda FJ, Blanca-Herrera RM, Agil A (2013). Antioxidant activity of melatonin in diabetes in relation to the regulation and levels of plasma Cu, Zn, Fe, Mn, and Se in Zucker diabetic fatty rats. Nutrition.

[23] Agil A, Rosado I, Ruiz R, Figueroa A, Zen N, Fernandez-Vazquez G (2012). Melatonin improves glucose homeostasis in young Zucker diabetic fatty rats. J Pineal Res.

[24] Catta-Preta M, Mendonca LS, Fraulob-Aquino J, Aguila MB, Mandarim-de-Lacerda CA (2011). A critical analysis of three quantitative methods of assessment of hepatic steatosis in liver biopsies. Virchows Arch.

[25] Gusdon AM, Fernandez-Bueno GA, Wohlgemuth S, Fernandez J, Chen J, Mathews CE (2015). Respiration and substrate transport rates as well as reactive oxygen species production distinguish mitochondria from brain and liver. BMC Biochem.

[26] Wolden-Hanson T, Mitton DR, McCants RL, Yellon SM, Wilkinson CW, Matsumoto AM, Rasmussen DD (2000). Daily melatonin administration to middle-aged male rats suppresses body weight, intraabdominal adiposity, and plasma leptin and insulin independent of food intake and total body fat. Endocrinology.

[27] Rasmussen DD, Boldt BM, Wilkinson CW, Yellon SM, Matsumoto AM (1999). Daily melatonin administration at middle age suppresses male rat visceral fat, plasma leptin, and plasma insulin to youthful levels. Endocrinology.

[28] Eckel RH, Depner CM, Perreault L, Markwald RR, Smith MR, McHill AW, Higgins J, Melanson EL, Wright KP (2015). Morning circadian misalignment during short sleep duration impacts insulin sensitivity. Curr Biol.

[29] Chojnacki C, Walecka-Kapica E, Klupinska G, Pawlowicz M, Blonska A, Chojnacki J (2015). Effects of fluoxetine and melatonin on mood, sleep quality and body mass index in postmenopausal women. J Physiol Pharmacol.

[30] Favero G, Stacchiotti A, Castrezzati S, Bonomini F, Albanese M, Rezzani R, Rodella LF (2015). Melatonin reduces obesity and restores adipokine patterns and metabolism in obese (ob/ob) mice. Nutr Res.

[31] Legros C, Devavry S, Caignard S, Tessier C, Delagrange P, Ouvry C, Boutin JA, Nosjean O (2014). Melatonin MT(1) and MT(2) receptors display different molecular pharmacologies only in the G-protein coupled state. Br J Pharmacol.

[32] Kemp DM, Ubeda M, Habener JF (2002). Identification and functional characterization of melatonin Mel 1a receptors in pancreatic beta cells: potential role in incretin-mediated cell function by sensitization of cAMP signaling. Mol Cell Endocrinol.

[33] MacKenzie RS, Melan MA, Passey DK, Witt-Enderby PA (2002). Dual coupling of MT(1) and MT(2) melatonin receptors to cyclic AMP and phosphoinositide signal transduction cascades and their regulation following melatonin exposure. Biochem Pharmacol.

[34] Sartori C, Dessen P, Mathieu C, Monney A, Bloch J, Nicod P, Scherrer U, Duplain H (2009). Melatonin improves glucose homeostasis and endothelial vascular function in high-fat diet-fed insulin-resistant mice. Endocrinology.

[35] Celinski K, Konturek PC, Slomka M, Cichoz-Lach H, Brzozowski T, Konturek SJ, Korolczuk A (2014). Effects of treatment with melatonin and tryptophan on liver enzymes, parameters of fat metabolism and plasma levels of cytokines in patients with non-alcoholic fatty liver disease--14 months follow up. J Physiol Pharmacol.

[36] Wang Z, Ni L, Wang J, Lu C, Ren M, Han W, Liu C (2016). The protective effect of melatonin on smoke-induced vascular injury in rats and humans: a randomized controlled trial. J Pineal Res.

[37] Allagui MS, Hachani R, Saidi S, Feriani A, Murat JC, Kacem K, El feki A (2015). Pleiotropic protective roles of melatonin against aluminium-induced toxicity in rats. Gen Physiol Biophys.

[38] Pan M, Song YL, Xu JM, Gan HZ (2006). Melatonin ameliorates nonalcoholic fatty liver induced by high-fat diet in rats. J Pineal Res.

[39] Hayakawa R, Hayakawa T, Takeda K, Ichijo H (2012). Therapeutic targets in the ASK1-dependent stress signaling pathways. Proc Jpn Acad Ser B Phys Biol Sci.

[40] Lawan A, Zhang L, Gatzke F, Min K, Jurczak MJ, Al-Mutairi M, Richter P, Camporez JP, Couvillon A, Pesta D, Roth Flach RJ, Shulman GI, Bennett AM (2015). Hepatic mitogen-activated protein kinase phosphatase 1 selectively regulates glucose metabolism and energy homeostasis. Mol Cell Biol.

[41] de Luxan-Delgado, Potes BY, Rubio-Gonzalez A, Caballero B, Solano JJ, Fernandez-Fernandez M, Bermudez M, Rodrigues Moreira Guimaraes M, Vega-Naredo I, Boga JA, Coto-Montes A (2016). Melatonin reduces endoplasmic reticulum stress and autophagy in liver of leptin-deficient mice. J Pineal Res.

[42] Xu L, Bai Q, Rodriguez-Agudo D, Hylemon PB, Heuman DM, Pandak WM, Ren S (2010). Regulation of hepatocyte lipid metabolism and inflammatory response by 25-hydroxycholesterol and 25-hydroxycholesterol-3-sulfate. Lipids.

[43] Glass CK, Olefsky JM (2012). Inflammation and lipid signaling in the etiology of insulin resistance. Cell Metab.

